# HIV Research Training Partnership of the University of Zambia and Vanderbilt University: Features and Early Outcomes

**DOI:** 10.5334/aogh.2588

**Published:** 2019-11-05

**Authors:** Wilbroad Mutale, Selestine Nzala, Holly M. Cassell, Marie H. Martin, Benjamin H. Chi, Mulenga Mukanu, Perfect Shankalala, John R. Koethe, Douglas C. Heimburger

**Affiliations:** 1 University of Zambia, Lusaka, ZM; 2 Vanderbilt University Medical Center and Vanderbilt Institute for Global Health, Nashville, Tennessee, US; 3 University of North Carolina, Chapel Hill, North Carolina, US

## Abstract

**Background:**

Despite the burden of HIV being highest in sub-Saharan Africa (SSA), research expertise and capacity to address scientific questions regarding complications of HIV and ART, especially chronic non-communicable conditions, is limited in the region. The comorbidities prevalent in persons with HIV are mediated through diverse mechanisms, many of which can be context or region-specific and are yet to be elucidated. The phenotype, risk factors, and effective interventions for these conditions may differ between populations and settings, and therefore there is an urgent need for research to help understand these processes and how to best address them in SSA. Here, we report the research capacity building activities in SSA conducted by the University of Zambia (UNZA)-Vanderbilt Training Partnership for HIV-Nutrition-Metabolic Research (UVP), drawing lessons and challenges for a wide global health audience.

**Methods:**

We reviewed program data and conducted interviews with program leaders and participants to understand and document the progress and outcomes of the partnership. We report the program’s early achievements, highlighting drivers and challenges.

**Results:**

Between 2015 and 2019, UVP made substantial progress on its goals of training new UNZA PhD scientists to investigate complex nutritional and metabolic factors related to long-term HIV complications and comorbidities. The program has supported 11 UNZA PhD students with dual UNZA-Vanderbilt mentorship; three have graduated, and other candidates are progressing in their PhD studies. The project also supported institutional capacity through UNZA faculty participation in Vanderbilt grant writing workshops, with strong success in obtaining grants among those who participated. UVP also supported development of greater structure to UNZA’s PhD program and a mentorship curriculum, both now adopted by UNZA. The major drivers for success included UVP’s alignment of goals between UNZA and Vanderbilt, and local institutional ownership. The longstanding history of collaborations between the two institutions contributed substantially to alignment and mutual support of UVP’s goals. Several challenges were noted, including limits on direct research funding for students and a relatively small pool of funded investigators at UNZA.

**Conclusions:**

Despite some challenges, UVP has achieved positive outcomes over its first four years. Longstanding partnerships and local institutional ownership were the main drivers. We expect the challenges to mitigated as the project continues and produces more UNZA researchers and teams and more funded projects, collectively building the local research community. With continued resources and clear focus, we expect that UNZA’s investigators and partners will attract research funding and generate high-impact research outputs across a broad range of studies in HIV as well as newer threats from non-communicable conditions experienced by long-term survivors of HIV and by the general population.

## Background

**Figure 1:**
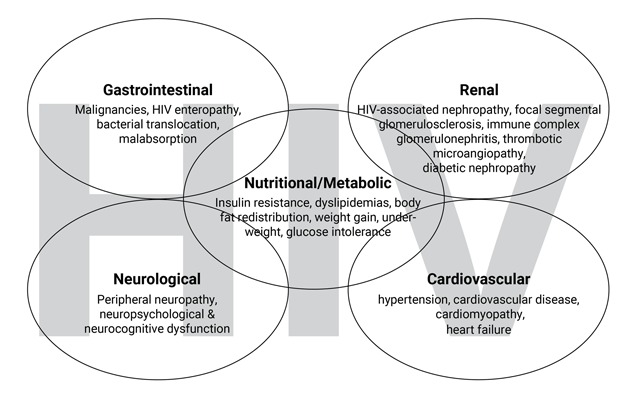
HIV related complications in different body systems.

The HIV pandemic has been a global health priority in recent years. The immense resources committed to addressing it have resulted in tens of millions of total life years gained, largely attributable to the success of antiretroviral therapy (ART) and the extensive programs designed to implement and monitor it [1]. While adults and children with HIV are now living longer, they face new challenges from long-term complications and comorbidities, including many non-communicable conditions. Despite the burden of HIV being highest in sub-Saharan Africa (SSA), research expertise and capacity to address scientific questions regarding complications of HIV and ART, especially chronic non-communicable conditions, is limited in SSA institutions [2]. Nutritional/metabolic factors are observed to be present in many individuals with long-term complications of HIV, including chronic comorbidities in the gastrointestinal tract, kidneys, endocrine, cardiovascular, and nervous systems (Figure 1) [3,4]. These complications are mediated through diverse biochemical processes, many of which are yet to be elucidated. Research is therefore needed to help understand these processes. Nutrition science and related scientific disciplines thus provide a strategic avenue for developing HIV research capacity in SSA while improving health outcomes.

Training partnerships have long contributed to building capacity in low- and middle-income countries (LMICs) [5]. The ongoing shift in the global development agenda from high-income countries (HICs) implementing programs in LMICs to a more collaborative approach has fueled the establishment of training partnerships between LMIC and HIC institutions. These partnerships can be at the individual level where grants are provided for training at master’s, PhD, or postdoctoral levels, or they can take the form of institutional support [6]. Institutional support grants, while fostering scientific collaborations, may provide funding to support research development through improvements in training facilities and research infrastructure, information and communication systems, and professional development opportunities for junior scientists and faculty members in the LMIC institution.

Several examples of capacity building activities exist but documentation of impact and sharing of transferable lessons have not been extensive, and consensus is lacking on the best approaches and how they should be reported [7,8,9,10].

**Figure 2:**
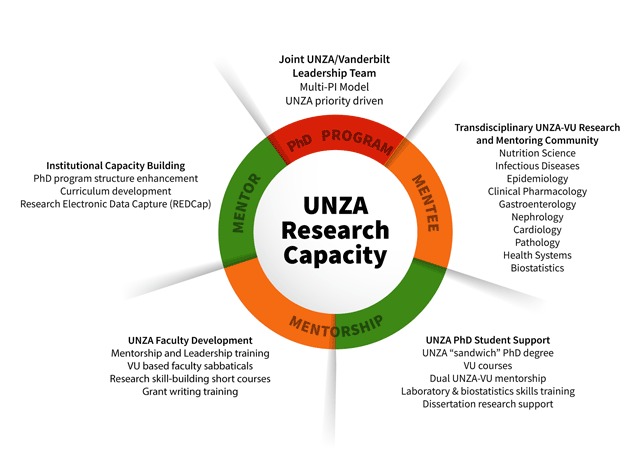
UVP Structure.

In order to build local capacity for advanced biomedical research knowledge and skills within the University of Zambia (UNZA) Ridgeway Campus (RWC) schools (comprising the schools of Medicine, Public Health, Health Sciences, and Nursing Sciences), the University of Zambia-Vanderbilt Training Partnership for HIV-Nutrition-Metabolic Research (UVP) was funded in 2015 by the Fogarty International Center (FIC) of the US National Institutes of Health (NIH), through 5-year grant D43TW009744 under PAR-13-126 [11]. The mission of UVP is to enhance the training of new PhD-level UNZA HIV research scientists, specifically in nutritionally- and metabolically-related complications and comorbidities of HIV. In particular, the training partnership aims to expand UNZA’s institutional research and training capacity through linkage to a top-tier US research institution, where strategic research skills are acquired by trainees and collaborative relationships established through structured, long-term educational rotations of Zambian PhD students at the US partner site. UVP was designed to advance UNZA’s strategic goals and respond to the growing need for research that addresses complications and comorbidities in HIV survivors and contribute to the government’s strategies of scaling up efforts to mitigate HIV/AIDS and Non-Communicable Diseases (NCDs). UVP is administered by a leadership team comprising senior faculty and administrators Vanderbilt University (VU) and UNZA who share responsibilities and decision-making (Figure 2). The team directs all day-to-day activities including program management, student mentoring and coordination, monitoring and evaluation, in-country trainings, curriculum development, selection processes, procedural standards, and progress reports.

In this report, we aim to document the progress of UVP to date and add to the body of knowledge on potential impacts of training partnerships on LMIC institutions’ research capacities. We hope that evaluation of our program outcomes may be helpful in improving and expanding future programs and partnerships.

## Methodology

### Study Design

We adopted a qualitative approach to report activities and lessons learned under UVP. This was a process review and documentation with the goal of sharing experiences.

### Data Extraction

We reviewed annual progress reports and activity reports and consulted with key program personnel. Generally, reports were limited to descriptions of an activity, output, or outcome but not impact, which will be accomplished with long-term follow-up. All descriptions were extracted and reviewed manually. We also interviewed some beneficiaries of the program to get their perspective and experiences on UVP.

The data were organized by UVP’s specific aims and relevant subthemes related to the aims. For consistency and shared learning, three research team members independently reviewed the reports and agreed on major themes and subthemes and content. When discrepancies were noted, these were discussed until consensus was reached.

### Data Synthesis and Analysis

The team reviewed all extracted material. We followed pre-determined themes based on project aims and objectives to report our findings. While we explored individual aims, we also focused on linkages across the aims. The findings were checked with other program staff to ensure the study reflected what was achieved and limitations. Their feedback helped to focus the analysis and to validate and interpret the results.

### Ethical Considerations

The study obtained exemption from the University of Zambia Biomedical Research Ethics Committee, as it was deemed evaluation of an existing university program.

## Results

We report achievements and progress according to UVP’s three specific aims.

### Aim 1. Train PhD Scientists from UNZA to Investigate Complex Nutritional and Metabolic Factors Related to Long-term HIV Complications and Comorbidities

UVP’s most intensive activity is to support UNZA PhD students whose research interests align with the focus of UVP, through a highly tailored and flexible program. Students first apply and are accepted into the UNZA PhD program, which requires a preliminary research proposal under the guidance of an UNZA mentor. Upon acceptance, students apply for support from UVP through an application developed in Vanderbilt’s Research Electronic Data Capture (REDCap) tool [12]. Applicants are evaluated principally on the alignment of their proposed research topics with the mission of UVP and the availability of suitable co-mentors at Vanderbilt, as a key concept in UVP’s design is interdisciplinary mentorship from investigators at both UNZA and Vanderbilt (Table 1).

**Table 1:** Details of UVP trainees.

Trainee	Current professional role	Study title	UNZA mentors	VU mentors	Progress

Male pharmacist	Lecturer, UNZA School of Health Sciences, and Head, Department of Pharmacy	The effect of lipid-based nutrient supplements on renal excretion of electrolytes and tenofovir concentrations in blood among Zambian HIV/AIDS patients	Nutrition scientist, pharmaceutical scientist	Pharmacologist, HIV/nutrition scientist	Graduated in 2016
Female physician	Lecturer, UNZA School of Medicine, affiliated with the Tropical Gastroenterology and Nutrition Group (TROPGAN)	Gastric cancer in Zambia: An assessment of associated factors and strategies for early case detection	Nutrition scientist, neurologist	HIV/nutrition scientist, cancer pathologist	Graduated in 2019
Male physician	Lecturer and Head, Division of Infectious Diseases, UNZA School of Medicine & University Teaching Hospital, Asst Director, Infectious Diseases, Ministry of Health, Adjunct Asst Professor, VU Department of Medicine.	Insulin resistance and systemic inflammation among HIV-positive, antiretroviral therapy (ART)-treated, virologically suppressed Zambian adults	Gastroenterologist (head of department), biostatistician	HIV/nutrition scientist, HIV infectious disease specialist	Graduation anticipated in 2019
Female biomedical scientist	Research Fellow, Centre for Infectious Disease Research in Zambia (CIDRZ)	Breast milk components and their effects on rotavirus vaccine seroconversion in Zambian infants born to HIV+ and HIV– mothers	Vaccinologist, basic scientist, nutrition scientist	Immunologist & molecular geneticist	Graduation anticipated in 2019
Male physician	Lecturer, UNZA School of Medicine, Nephrology Coordinator, Zambia Ministry of Health	Renal and nutritional biomarkers in tenofovir induced nephropathy among HIV infected adolescents and young adults at UTH, Lusaka, Zambia	Nutrition scientist	HIV/nutrition scientist, HIV infectious disease/nephrologist	Graduation anticipated in 2020
Male biomedical scientist	Lecturer, School of Health Sciences, UNZA	Probabilistic baseline prediction of TDF-associated nephrotoxicity among ART-naïve HIV-infected adults in Zambia	Pharmaceutical scientist, HIV/infectious diseases specialist, nephrologist (all UVP alumni)	Nephrologist, HIV infectious disease specialist, HIV/nutrition scientist	Graduation anticipated in 2020
Female physiology lecturer	Lecturer, Lusaka Apex Medical University and Head, Department of Human Physiology	Endothelial dysfunction and plasma peroxynitrite levels in human immunodeficiency virus (HIV) infected individuals in Zambia	Cardiovascular physiologist, infectious diseases specialist	HIV infectious disease specialist, endothelial lipid peroxidation biologist	Graduation anticipated in 2020
Female physiology lecturer	Lecturer, Cavendish University Zambia, School of Medicine	Endothelial dysfunction and dysglycemia in lean HIV+ adults on ART	Cardiovascular physiologist	HIV infectious disease specialist, inflammation/hypertension scientist	Graduation anticipated in 2021
Male epidemiologist	Lecturer, Mulungushi University School of Medicine and Health Sciences	Predictors of virological failure among HIV patients with metabolic syndrome receiving antiretroviral therapy in Livingstone, Zambia	Epidemiologist, public health scientist, biostatistician	NCD epidemiologist, HIV/nutrition scientist	Graduation anticipated in 2021
Male biochemist	Clinical Biochemist and Lecturer, Mulungushi University School of Medicine and Health Sciences	Patho-immune mechanisms of hypertension in HIV-infected patients	Infectious disease immunologist, molecular microbiologist	Inflammation/hypertension scientist, HIV infectious disease specialist	Graduation anticipated in 2021
Male health economist	Lecturer, UNZA, School of Public Health	Health inequality among hypertensive patients in Zambia, stratified by HIV status	Health economist, health systems scientist	Behavioral economist, health care economist	Beginning PhD studies

UVP’s support is organized in a “sandwich” fashion, in which students come to Vanderbilt early in their first PhD year (except in one case, the University of Cape Town, South Africa) to take courses tailored to their specific interests and requirements for new skills, to solidify relationships with mentors, to identify additional Vanderbilt mentors where needed, and to work with their UNZA and Vanderbilt mentors to refine their research proposals. Courses taken have included biostatistics, research ethics, epidemiology, clinical trials, genetics, immunobiology, bioregulation, microeconomics, and analysis of survey data. Additionally, UVP trainees join the community of international scholars at Vanderbilt Institute of Global Health (VIGH) as well as the diverse international student community at Vanderbilt.

After two to four months at Vanderbilt, the trainees return to Zambia where they apply for approval of their PhD thesis research proposals by the UNZA Board of Graduate Studies and the UNZA Biomedical Research Ethics Committee. In years two and three, they typically collect their research data with modest research cost support from UVP. In year three or four, they may return to Vanderbilt for additional mentorship and training including data analysis, manuscript preparation, and grant writing. To date, all biological assays on collected specimens have been conducted in Zambia, but students may transfer samples to Vanderbilt if the required technology is not feasible in Zambia. In year four, they write and defend their theses orally (*viva voce*) and submit manuscripts for publication.

Eleven PhD students have received sponsorship from UVP thus far (Table 1). All of them have continued their primary occupations while pursuing PhD studies: six of them teach at UNZA (three of these are also physicians at the University of Zambia Teaching Hospital [UTH]), four teach at Zambian universities other than UNZA, and one is a research fellow at a local research institute. Below are some observations by UVP fellows on how the program helped and some challenges:

***[Benefits and gaps filled by UVP]***

*“I have been sponsored for tuition, trip to the USA (twice) for enhancement courses, had one on one mentorship…have had opportunity to present ideas to a broader community in Zambia and the USA.” [Most important gaps filled by UVP] “Exposure to more ways of handling research, statistics, and clinical investigation research and mentorship.”* ***UVP Fellow_1***

*“The clinical trial course I took at Vanderbilt was so key to the development of my research focus area and the Vanderbilt trip was an eye open to a number of things pertinent to career development*.” ***UVP Fellow_7***

***[Mentorship experience]***

*“I have a well-rounded team with my primary supervisor being my main mentor and I have also received great mentorship from the Vanderbilt team.”* ***UVP Fellow_2***

*“I feel comfortable and well equipped with a flexible, serious, and helpful mentorship team. I have received all the necessary help. My mentorship team is multidisciplinary and this has been helpful in tackling various issues I had.”* ***UVP Fellow_5***

*“I have a great mentorship team with different expertise which are all cardinal to my career path. My mentors are always there when I need help.”* ***UVP Fellow_8***

***[Challenges faced by fellows]***

*“Given that we are doing PhDs, the financial amount for research is not enough to run the project smoothly. There is a limitation to what one can explore hence having some potential to affect (to some degree) the impact of the research. Otherwise, it has been a successful journey and program.”* ***UVP Fellow_4***

*“My main challenge has been integrating work and research time…. I did not know what to expect at Vanderbilt. I wish I had used the resources better at Vanderbilt if I had prior arranged time in the lab to learn techniques and have more practice time.”* ***UVP Fellow_7***

*“The research money is not very enough for someone to do all the lab tests deemed important in answering the aims. However, the project is running smoothly generating results of public health and policy importance.”* ***UVP Fellow_2***

*“I have not been able to obtain all the required online learning materials on time due [to] internet challenges.”* ***UVP Fellow_8***

### Aim 2. Strengthen the UNZA PhD Program to Enhance Its Capacity to Train Future Researchers, Attract Research Funding, and Generate High-impact Research Outputs Across a Broad Range of Studies

#### Review and revision of UNZA PhD program

Because UNZA RWC leaders and faculty members expressed interest in reviewing the PhD Program structure and curriculum to identify opportunities for improvement, we conducted a PhD Program needs assessment and evaluation in May 2016. We reviewed UNZA training and policy documents and conducted individual in-depth interviews and group consultations with key stakeholders including administrators, faculty advisors, students, and alumni. We examined domains used in US academic program reviews that are designed to improve effectiveness and increase academic quality, including learning outcomes, teaching and learning, mentorship, student learning assessment, research environment, and administration and support.

Recommendations that emerged from the assessment included developing a shared advanced research methods course for all schools and departments in the RWC that could maximize limited resources, leverage faculty expertise, and address a common need. Additionally, the creation of program-specific didactic courses was recommended to ensure that students would have strong foundations in their fields for research, teaching and career advancement. Recognizing the importance of mentorship in academic success, research productivity and retention, the enhancement and formalization of mentor training and improved faculty mentorship resources were advised. Recommendations also included establishment of a PhD Program Director and Steering Committee to improve the program’s administrative processes. A formal report of the assessment was generated and disseminated to UNZA RWC leadership.

Responses to the report’s dissemination revealed a collective aspiration among administrators, faculty, and students to have a strong and competitive PhD program at UNZA, and consensus around the need for a structured curriculum. As a result of the assessment, UNZA formed a PhD Program Committee to develop a road map that was subsequently approved by the UNZA Board of Graduate Studies, and efforts are underway to identify and develop the courses required to complete the structured curriculum.

#### Enhancing mentorship in the UNZA PhD program

In response to the request for stronger PhD student mentorship, the UVP teamed with the *University of North Carolina (UNC)-UNZA-Wits Partnership for HIV and Women’s Reproductive Health (UUW)* (NIH/FIC) [13] to develop a mentorship curriculum at UNZA through an FIC Clayton-Dedonder Global Health Mentorship Fellows Program grant supplement [14]. The aims of the program were to 1) design and implement a PhD mentor training program for all four schools at UNZA RWC, 2) train ten or more UNZA Mentoring Fellows to mentor UNZA PhD students, and 3) use an iterative evaluation model to optimize the training program and integrate it as a required component of the UNZA PhD Program.

The mentorship curriculum was developed as a two-tiered capacity building program, engaging ten senior UNZA faculty members to hone their mentorship skills by designing and pilot testing a curriculum to train ten junior UNZA faculty mentors. Dyads of senior UNZA faculty members led the curriculum development process to ensure context-appropriateness and sustainability. Vanderbilt and UNC supported the curriculum design and implementation by leveraging cutting edge educational approaches and resources. The curriculum was implemented from June to October 2018 at UNZA through a series of five full-day workshops spread over five months, led by UNZA facilitators utilizing interactive instructional strategies and digitally accessible materials. Prior to each workshop, participants were expected to complete background readings and assignments for the ten select topical modules. In addition to building durable competencies in effective mentorship and leadership, a goal of the program is to transform the institution’s culture of mentorship.

During the implementation of the pilot program, quantitative and qualitative data were collected at multiple points through process and impact evaluations. A variety of instruments including pre- and post-assessments and workshop and program evaluations were used to assess program development and implementation. An additional culture survey was administered before and after the program to measure changes in UNZA’s mentoring culture. Participant evaluations provided opportunities for quality improvement of presentations, activities, assessment, and logistics. The process evaluations revealed that the curriculum development process contributed to increased interest, knowledge, and self-efficacy in new educational strategies among facilitators. Impact evaluations, including a pre- and post- mentorship competency assessment, program evaluation, and workshop pre- and post-tests, are currently underway and results will be published separately. Below are some quotes from those who took part in the mentorship training.

***[Impression of mentorship training]***

*“Mentorship is a holistic interaction which goes beyond a specific subject area. It aims to bring about independence, critical thinking, and professional development in the mentee through academic leadership of mentor…we have started implementing the taught information with students in our research group and we try to move ourselves from being supervisors to being mentors.”* ***UVP Junior Mentorship Fellow***

*“[This was a] Mind opening experience. A lot of learning for me. I think it is very useful and it has come at a time when UNZA really needed to start such a programme.”* ***UVP Senior Mentorship Fellow***

*“The mentoring program has given me a lot of strength in mentorship. I have been a teacher a long time but not a mentor. Now I am a mentor teacher, whereas I was a ‘teacher teacher’ before. Mentors are teachers but not all teachers are mentors. It gave me big insight into shaping junior colleagues in the profession. I am now looking out for people to mentor. I’m going back through the materials and learning more. When I went through the materials during the program, I was assimilating them for the course, but now I’m going through them again and understanding them much more. I’m ready to be a curriculum designer now. There is a different dimension to my teaching. It’s been very capacity building, and resources were very well spent. I made sure that I didn’t miss one session.”* ***UVP Senior Mentorship Fellow***

*“Having undergone the mentorship programme, I now understood that mentorship is a discipline with its own established guiding principles. Prior to the program, I had no personal philosophy, now I have one written down ‘excellence in performance, attitude and outlook’. Based on the principle of aligning mentee/mentor relation, I have revisited my mentorship strategy. At the school level I have started advocating for structured mentorship.”* ***UVP Junior Mentorship Fellow***

*“The University of Zambia was anchored on the art of supervising the students and rarely used the mentoring word. These mentorship training sessions were key to assuming the role of strong mentor.”* ***UVP Senior Mentorship Fellow***

#### Annual research workshops

UVP’s Aim 2 includes annual research workshops held at UNZA to discuss the latest science and to build skills relevant to UVP’s research focus and UNZA research opportunities. To date, 250 persons have participated in the following workshops:

- Nutrition and NCDs: Knowledge Gaps and Burning Issues, January 2016
- Methods for Research in HIV, Nutrition, and Metabolism, March 2017
- Cancer Research in Zambia: Programs, Progress, and Opportunities, April 2018
- Cardiovascular Diseases Research in Zambia: Programs, Progress, and Opportunities, February 2019

Each workshop topic was selected by the UVP leadership team in consultation with local researchers, and showcase research completed in or underway in Zambia, principally conducted by UNZA faculty members, and research conducted by speakers visiting from Vanderbilt and other Southern African and European countries. All current UVP-supported trainees also Provided plenary-session updates on the progress of their research, and thus received input on their research from workshop attendees.

#### Grant Writing training

VIGH’s annual month-long grant writing workshop, the Vanderbilt Institute in Research Development & Ethics (VIRDE), was developed in 2011 solely for VIGH’s LMIC partners, with support from the AIDS International Training and Research Program (AITRP) grant that preceded UVP. It has expanded to include scholars supported by other VIGH-managed FIC training grants and by the Doris Duke Charitable Foundation. VIRDE provides intensive training in research development, productivity, and ethics, and is intended to help investigators develop the skills necessary to conduct responsible human subjects research and develop a grant proposal for submission. VIRDE provides mentoring in grant writing through dedicated VU and in-country mentor teams who meet with trainees several times each week, plus more than 30 contact hours of specially tailored seminars in grant writing and research ethics and integrity. Since 2011, 45 LMIC health professionals engaged in research in their home countries, including Fogarty research training grant alumni, have gained fundamental skills in developing grant proposals to fund their work. Of these, 16 have been UNZA RWC faculty members supported by the AITRP and UVP grants. A full evaluation for the VIRDE program is currently underway and will be reported elsewhere.

#### Career development opportunities for research-focused UNZA faculty members

Also, in support of Aim 2, UVP sponsors four-month VU-based sabbaticals for UNZA faculty members whose research focus aligns with UVP’s. So far, two faculty have taken sabbaticals at VU; outcomes have included new collaborative relationships, grant applications, and publications that would not have been possible without the protected time afforded by UVP support.

### Aim 3. Ensure and Document the Long-term Success of the Training Program (Evaluation Plan)

**Figure 3:**
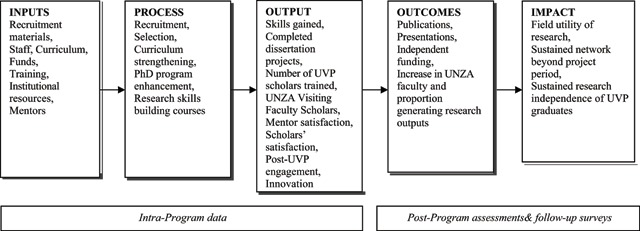
UVP Evaluation Plan.

We evaluate UVP using a mixed-method approach including process, outcome and impact (see Figure 3). Process evaluations are routinely conducted to ensure that we meet program objectives and to identify opportunities for program process improvement. For example, the first cohort of UVP applicants was solely from the School of Medicine, so we needed to evaluate how to broaden our reach across the schools. Through an iterative process, evaluation, advertisement, recruitment, and selection procedures were streamlined in the second year to provide a broader reach across the four RWC schools. This was effectively actualized in the remaining UVP PhD trainee cohorts. Additionally, we routinely conduct evaluations assessing trainee progress toward PhD degrees to ensure timely completion; trainees’ and mentors’ perceptions of the quality of the mentor support to ensure that mentor-mentee relationships are productive; and exit interviews of UVP trainees and mentors to enhance program strengths and identify areas for improvement. Anonymous online program evaluations are conducted at the completion of all short courses to assess skills enhancement and overall opinions about the courses. Each course evaluation informs the development of subsequent courses.

#### Monitoring and evaluating the impact of trainee-generated research and capacity building

Concrete research career outcomes are collected annually by extracting applicable career measures such as publications, post-training presentations, and grant proposals submitted and funded. Data are entered in the NIH CareerTrac system. Key benchmark measures include UVP trainee and UNZA Faculty Scholar research productivity, leadership, participation in global research networks, and overall research contribution and impact including discoveries made and trainees mentored by UVP alumni. Because most of the UVP-supported students have not yet graduated, their productivity of publications, grant applications, and other measures of local and global impact is yet to be realized. However, thus far, UVP scholars have generated 27 publications, eight oral presentations at global conferences, and two funded grants. Through a process of ongoing monitoring and evaluation of individual and programmatic output, there is an intention that the training activities facilitated by UVP align with the research training needs identified by UNZA and yields results that accrue to the health of the Zambian people, with potential for dissemination throughout the sub-Saharan African region.

#### Collaborative UNZA-Vanderbilt research community

An overarching goal of UVP is to build a community of researchers at UNZA with collaborations with VU investigators. While at VU, UVP trainees engage substantially with mentors from various VU departments toward this end, and they have been openly welcomed by individuals and research groups from Internal Medicine (Infectious Diseases, Nephrology, Translational and Clinical Cardiovascular Research Center, Epidemiology, and others), Pharmacology, Cell Biology, Vascular Biology, and Biostatistics, and from VU Master of Science in Clinical Investigation Program and Interdisciplinary Graduate Program and VU’s neighbor institution, Meharry Medical College.

The UNZA research community is envisioned as comprising both UNZA PhD graduates and their mentors who have participated in a shared UVP-supported research training process, have built collaborations with Vanderbilt investigators, [15,16,17,18,19,20,21,22] and are pursuing research careers focused on nutrition/metabolism-related long-term non-communicable complications and comorbidities of HIV. It will take time for these communities to mature, but there is concrete early evidence of it, including UVP students who are farther along in their careers and UVP alumni co-mentoring trainees who are earlier in their studies, and publications and grant applications being submitted by UNZA/Vanderbilt mentor-mentee teams that have convened through UVP support.

## Discussion

### Importance of High-level Support and Engagement at Program Initiation

UVP was designed to engage with senior management at both institutions to ensure their input on the priorities for the collaboration, especially gaps that could be bridged to catalyze future research collaborations. The focus on capacity building was therefore based on consensus and priorities for the University of Zambia. Past collaborations also informed this initiative, as very little attention had been paid to doctoral training and faculty support, which were major gaps. With this in mind, both senior and junior faculty members have been capacitated to co-mentor and supervise PhD students through this initiative. UNZA senior administration has taken much interest, including a high-level visit of UNZA administrators to Vanderbilt to show support and encourage the collaboration. The high-level involvement has resulted in shared vision and commitment. For example, processes to recruit PhD program graduates into full-time positions at UNZA and other collaborating local universities have been streamlined. UNZA is also implementing a plan to integrate the PhD curriculum plan and the mentorship curriculum supported by UVP into the university system as part of UNZA’s standards and requirements.

### Co-mentoring for PhD Fellows and Capacity Exchange

UVP aims to build capacity not just for students but for UNZA faculty as well. Senior UNZA faculty were deliberately paired with those at Vanderbilt, creating a shared vision for mentorship and expanding opportunities for mentors from both institutions. The Vanderbilt faculty were selected based on their special expertise that aligned with the students’ research interests. Students share their progress regularly with all mentors, leading to collegial interchanges with input from both sides. The UNZA mentors understand the field-work logistics and PhD standards at UNZA, a combination that provides scientific strength and general research capacity and innovation. The PhD trainees’ tenures at Vanderbilt at the beginning and end of their PhD studies provide them with both intensive international mentorship and peer learning. Some of these groups have extended their collaborative activities beyond the PhD training, evidencing the communities of researchers we wish to create.

### Institutional Capacity Building

While UVP’s Aim 1 focuses on individual PhD students and junior faculty members, institutional research capacity building is the program’s overarching objective. In the annual research workshops, UNZA faculty members present their research findings along with and aligned with student research presentations. This was the first regular platform at the University of Zambia in which research could be shared and themes chosen by faculty members each year. The platform also allows for internationally renowned scientists, including those from other regional institutions, to share their research with UNZA faculty and students as invited guests. Another institution-level activity is the collaborative development of the mentorship training curriculum which is being adopted as standard for training all PhD mentors at UNZA RWC. Moving forward, it is hoped these activities will be locally owned and extended to other schools.

### Leveraging Existing Structures and Opportunities at UNZA

UVP utilizes UNZA resources that had been introduced through previous projects and collaborations, including prior extramurally funded research training programs such as NIH AITRP grants. For example, faculty trained through previous grants are the main resource used to support current PhD students and UVP’s activities. Selected existing UNZA courses have been identified and offered to UVP-supported PhD students, and are now being modified to enhance the education of all UNZA PhD students. Additionally, both previous and ongoing studies, cohorts, and specimen and data banks are being utilized by PhD students to conduct their research.

These synergies have been realized at a lower cost than that required to start such a program from scratch and have emphasized the need for long-term investments in research training and collaboration. UVP’s achievements were built on the foundation of previous gains that might have gone unnoticed yet are vital to success in building LMIC institutions’ research capacity.

### The Role of Technology

UVP has benefitted from advances in technology that support learning. Students and faculty have utilized platforms like WhatsApp to communicate and share ideas rapidly. Zoom, Skype, and GoToMeeting have been used for fortnightly leadership meetings as well as trainings, webinars, and mentorship sessions. This has helped overcome barriers associated with distance, costly telephone calls, and logistics of local and international travel. Technology is supplemented with physical mentorship meetings and workshops, thus making the program seamless and efficient despite the distance between the US and Zambia.

Research Electronic Data Capture (REDCap) is an additional technological resource that has been instrumental in UVP students’ acquisition and management of data for their research projects [11]. Invented and managed by scientists at Vanderbilt’s Department of Biomedical Informatics, REDCap is a secure web application for building and managing online surveys and databases. REDCap was made available to UVP-supported students upon enrollment. UVP resources and workshops facilitated the installation and implementation of REDCap at UNZA, and it is anticipated that UVP will support training in its use as well, as a resource for investigators throughout the institution.

### Challenges and Limitations

As a research training program supported by a grant that has an NIH-imposed cap on research costs, UVP’s major challenge has been the modest level of funding available to support robust PhD student research projects. This, coupled with the modest number of ongoing Zambia-based research projects and UNZA-based research mentors that PhD students can join, has restricted the scope of the students’ biomedical and public health research. Given these circumstances, it is vital to have complementary research funding to enable students and faculty to conduct advanced and rigorous research. This mirrors the challenges of LMIC institutions generally [23,24]. The goal over time is to expand collaborations that enhance training and mentoring of program graduates in grant writing in order to obtain independent research funding. This promises to advance LMIC research generally and to strengthen the institutional fabric of mentoring and training at UNZA.

Similarly, the program has a stipend budget to support the students only while they are in the US; while UVP supports their tuition at UNZA to keep them enrolled in the PhD Program, it does not provide stipends while they are in Zambia. This affects students’ progress, as they must maintain employment to have adequate incomes, but it enables us to support more students.

Finally, high-speed internet access and access to some software resources at UNZA have been a challenge for students. While they have reasonable access to software through Vanderbilt, it will end when they are no longer students, so we are seeking local support for online resources to keep the PhD graduates competitive on the global scene.

As UVP has completed only four years, we have focused here on its features and short-term outcomes. We will report long-term impacts in a future publication after more trainees have graduated and their outcomes have matured.

## Conclusions

In its first four years, the UNZA-Vanderbilt Training Partnership for HIV-Nutrition-Metabolic Research has made substantial progress on its goals of training new UNZA PhD scientists to investigate complex nutritional and metabolic factors related to long-term HIV complications and comorbidities and strengthening UNZA RWC PhD Program’s capacity to train researchers. This paper documents both its progress and challenges to date. UVP’s existence and impact depend on a foundation established by UNZA and international partners, grounded in the multilateral effort to address the threat of HIV and with support from prior research training grants. These experiences suggest that with continued resources and clear focus, UNZA’s investigators and partners will attract research funding and generate high-impact research outputs across a broad range of studies in HIV as well as newer threats from non-communicable conditions experienced by long-term survivors of HIV and by the general population.

## Additional File

The additional file for this article can be found as follows:

10.5334/aogh.2588.s1 UNZA Mentorship. Programme Interview Guide.
