# Supplementary material for: HIV Research Training Partnership of the University of Zambia and Vanderbilt University: Features and Early Outcomes
Source: Ann Glob Health. 2019 Nov 5;85(1):129. doi: 10.5334/aogh.2588 (PMC6838769; doi:10.5334/aogh.2588)
Supplement: UNZA Mentorship. — Programme Interview Guide. [file agh-85-1-2588-s1.pdf]

UNZA Mentorship
Programme Interview Guide

The same script and guiding questions will be used for both individual interviews and focus groups.

Opening script: Hi, how are you today? Thank you for your time. As a reminder, I serve as the Principal Investigator for the UNZA Mentorship Programme evaluation study. This study is part of a larger evaluation study of the UNZA Mentorships Programme. With your permission, I will be recording this conversation solely for research purposes. The contents of this conversation will remain confidential and any identifiable information will be de-identified before analysis. There are no foreseen risks or threats to participating in this project and you can leave at any point. Are you comfortable with recording this conversation and proceeding forward? (Assumed “yes”.) Thank you. I have five main questions to guide our conversation and we should be finished in about (for focus groups) an hour / (for interviews) 25-30 minutes. Do you have any questions before we begin?

Guiding questions (see following pages, with room for notes)
**Note to self: Remember to ask clarifying questions such as “Do I understand that you mean…?”*

Closing script: Those are all the questions that I have. Thank you for your valuable insights! Do you have any questions for me about this conversation/discussion or the study? (Pause, wait for participant to answer, discuss any lingering questions.) Once again, thank you for your time. If you need to contact me for any reason, I am available by email at elizabeth.rose@vumc.org. Thank you and have a great rest of the week!

<u>Guiding questions for senior faculty trainers:</u>

1. Please describe your participation in the UNZA Mentorship Programme.

<u>Guiding questions for senior faculty trainers:</u>

2. Related to curriculum development and teaching methodology…
    a. Please describe key concepts, ideas, skills, and knowledge that you learned in the programme. What is the most powerful “take-away” or “lesson learned” from this experience?

    b. Please tell me about ways that you may have used any of the ‘learnings’ that you just described.
        i. Are there any ‘learnings’ that you haven’t begun to use but plan to implement in the future?
        ii. In what ways has your teaching changed as a result of the programme?
    c. Workshop presentations on teaching strategies, participating in workshop activities, and calls and meetings with Dr. Martin were methods used to introduce ideas in curriculum development and teaching methodology. Describe the utility of these in helping you learn

<u>Guiding questions for senior faculty trainers:</u>

3. Related to mentoring…
    a. Please describe key concepts, ideas, skills, and knowledge that you learned in the

Mentorship Programme. What is the most powerful “take-away” or “lesson learned” from this experience?

b. Please tell me about ways that you may have used any of those ‘learnings’ as you mentor students.
    i. Are there any ‘learnings’ that you haven’t begun to use but plan to implement in the future?
c. Developing the curriculum and presentations, participating in workshops, and reading workshop materials were methods used to introduce ideas in mentoring. Describe the utility of these in helping you learn

d. In what ways has your mentoring changed as a result of the programme

Guiding questions for senior faculty trainers:

4. What impact, if any, do you believe this programme has had at UNZA’s College of Medicine, particularly related to PhD students?

5. Is there anything else that you would like to discuss about the UNZA Mentorship Programme, your participation, the impact that it has had on your teaching and mentorship, or the impact that it has had on others in the College?

Guiding questions for mentor fellows:

1. Please describe your participation in the UNZA Mentorship Programme.

Guiding questions for mentor fellows

2. Please describe key concepts, ideas, skills, and knowledge related to mentoring that you learned in this programme. What is the most powerful “take-away” or “lesson learned” from this programme?
    a. Please tell me about ways that you may have used any of those ‘learnings’ as you mentor students.
    b. Are there any ‘learnings’ that you haven’t begun to use but plan to implement in the future
    c. In what ways has your mentoring changed as a result of the programme?

Guiding questions for mentor fellows

3. The workshops used a variety of teaching strategies to introduce mentoring concepts.
    a. Describe the utility of the readings and associated presentations and discussions in helping you learn.
    b. Describe the utility of the PowerPoint presentations in helping you learn.
    c. Describe the utility of small group activities and discussions in helping you learn.
    d. Describe the utility of case studies in Workshops 1 and 2 in helping you learn.
    e. Describe the utility of assignments, including the Leadership Philosophy and the Mentorship Philosophy in helping you learn.
    f. Describe any other element of the programme that you found particularly useful (or not useful) in helping you learn.
    g. What elements should definitely be kept in the programme
        i. What could be left out?
    h. What suggestions do you have to improve this Programme in the future?
        i. Should it be implemented in a different time frame (i.e. 1-week intensive course, spread out over one year, etc

UVP Fellow Evaluation questions:

Thank you for accepting to participate in the UVP evaluation. We will ask you questions about your experience please be as honest in answering the questions.

1. When did you become Fellow with UVP?
2. What were your expectations when you become a fellow?
3. What has been your experience so far?
4. What has been the most important gap the UVP has addressed in your career?
5. How to you describe your mentorship team?
6. What are some of the challenges you faced as a fellow in UVP?
7. What would be your recommendations to the UVP leadership team to further improve the programme?
